# Supplementary material for: ATP-powered molecular recognition to engineer transient multivalency and self-sorting 4D hierarchical systems
Source: Nat Commun. 2020 Jul 21;11:3658. doi: 10.1038/s41467-020-17479-9 (PMC7374688; doi:10.1038/s41467-020-17479-9)
Supplement: Supplementary file 1 — Supplementary Information [file 41467_2020_17479_MOESM1_ESM.pdf]

Supplementary Information for

ATP-powered molecular recognition to engineer transient multivalency and self-sorting 4D hierarchical systems

Jie Deng[1,2,3,4,5] *and Andreas Walther*[1,2,3,4,5]*

[1] A^{3}BMS Lab, Institute for Macromolecular Chemistry, University of Freiburg, Stefan-Meier-Straße 31, 79104 Freiburg, Germany

[2] DFG Cluster of Excellence "Living, Adaptive and Energy-Autonomous Materials Systems" (*livMatS)*, 79104 Freiburg, Germany

[3] Freiburg Materials Research Center, University of Freiburg, Stefan-Meier-Straße 21, 79104 Freiburg, Germany

[4] Freiburg Center for Interactive Materials and Bioinspired Technologies (FIT), University of Freiburg, Georges-Köhler-Allee 105, 79110 Freiburg, Germany

[5] Freiburg Institute for Advanced Studies (FRIAS), University of Freiburg, Albertstraße 19, 79104 Freiburg, Germany.

Andreas.walther@makro.uni-freiburg.de.

ssDNA single-stranded DNA

dsDNA double-stranded DNA

ATP adenosine triphosphate

WU Weiss Unit

AGE agarose gel electrophoresis

nt nucleotide

bp base pair

kbp kilo base pair

SfNAP sequence-defined functionalized nucleic acid polymer

DySS dynamic steady state

FRET Forster resonance energy transfer

DoS$_1$ docking strand$_1$

DoS$_2$ docking strand$_2$

TCA transient colloid assembly

TSP transient surface polymerization

CLSM confocal laser scanning microscopy

TSeSo transient self-sorting

Supplementary Methods

Instrumentation: ThermoMixer (Eppendorf), U:Genius^3 Gel electrophoresis documentation system (Syngene), UVsolo *touch* Gel electrophoresis documentation system (Analytik Jena), gel electrophoresis chambers (biostep), power source 250 V (VWR), Image J, ScanDropR UV-VIS spectrometer (Analytik Jena), QE *Pro* spectrometer with fluorescence measurement set-up (Ocean Optics), Mightex LED (λ_{exc} = 530 nm, 3 W, output: 270 mW; fiber coupled LED light source), CUV-QPOD temperature-controlled cuvette holder qpod 2e™ (Ocean Optics), Confocal laser scanning microscopy (Leica TCS SP8, Mannheim).

Reagents: T4 DNA Ligase (HC, 20 WU µL^{-1}, recombinant *E. coli* strain) was supplied by Promega and BsaI-HF®v2 (20 units µL^{-1}, *NEB #R3733*) was ordered from New England Biolabs (*NEB*). ATP solution (10 mM in 1 mM Tris-HCl pH 7.5) was purchased from Invitrogen. Agarose low EEO was supplied by PanReac AppliChem. Gene ruler 1k bp (SM0311) and 50 bp (SM0373) DNA ladders (ready to use) and DNA gel loading dye (6×) were ordered from ThermoFisher Scientific. Streptavidin coated magnetic microparticles, Dynabeads™ MyOne™ Streptavidin C1, were supplied by ThermoFisher Scientific. Ethylenediaminetetraacetic acid disodium salt dihydrate (EDTA, biology grade) was supplied by CALBIOCHEM. Sodium chloride (NaCl, 99%) and dodecane (99%) were ordered from ABCR. Tris (hydroxymethyl)aminomethane hydrochloride pH 8.0 (Tris-HCl) and trizma base were ordered from Sigma-Aldrich. Acetic acid glacial (ACS, Reag. Ph. Eur. Analytical reagents) was supplied by VWR Chemicals. RotiR-GelStain (1,1'3,3',5,5'6,6'-Octamethyl-2,2'-spiro(2,3-dihydro-1H-Benzimidazol) was supplied by Carl Roth.

Buffer compositions

T4 DNA Ligase Storage Buffer (Promega): 10 mM Tris-HCl (pH 7.4 at 25 °C), 50 mM KCl, 1 mM dithiothreitol (DTT), 0.1 mM EDTA, 50% glycerol.

BsaI-HF®v2 storage buffer (NEB): 10 mM Tris-HCl, 200 mM NaCl, 1 mM DTT, 0.1 mM EDTA, 200 µg mL^{-1} BSA, 50% glycerol.

NEB CutSmart® Buffer: 50 mM potassium acetate, 20 mM Tris-acetate, 10 mM magnesium acetate, 100 µg mL^{-1} BSA.

Annealing Buffer: 10 mM Tris-HCl (pH 8.0), 50 mM NaCl.

TAE Buffer: 40 mM Tris, 20 mM acetic ccid, 1 mM EDTA.

Quenching Buffer: 200 mM EDTA, 10 mM Tris-HCl (pH 8.0), 50 mM NaCl.

CSF buffer: *CutSmart® Buffer* supplemented with 0.05% Pluronic® F-127.

Milli-Q water was used throughout of this study.

Oligonucleotides: All oligonucleotides were supplied by Integrated DNA Technologies Inc.. The biotinylated DNA strands were ordered with standard desalting purification, and all the other DNA strands were ordered with HPLC purification.

Supplementary Table 1: Oligonucleotide sequences used, with their name in individual Figure, the sequence codes used for ordering at IDT, and their ID in experimental section.

	Name	Oligonucleotide sequence (5'-3')	ID
**Figure 2**	M1	/5Phos/AATCCTCTATTCGCATGAGAATTCCATTCACGGTCTCT	D_{1}
		/5Phos/GATTAGAGACCGTGAATGGAATTCTCATGCGAATAGAG	D_{2}
**Figure S2**		GTATTCCATTCACGGTCTCTAATCCTCTATTCGCATGAGATACG	D_{3}
		CGTATCTCATGCGAATAGAGGATTAGAGACCGTGAATGGAATAC	D_{4}
**Figure 3a**	A	CATGAGAATTCCATTCACGGTCTCT	D_{5}
		/5Phos/GATTAGAGACCGTGAATGGAATTCTCATG	D_{6}
	B	/5Phos/AATCCTCTATTCGCATGAGAATTCCATTCACGGTCTCT	D_{7}
		/5Phos/ATGAAGAGACCGTGAATGGAATTCTCATGCGAATAGAG	D_{8}
	C	/5Phos/TCATCATACTCAGACATCGTCAAGCTTTCTATTCGCATCAGAGGTCTCT	D_{9}
		/5Phos/GTCAAGAGACCTCTGATGCGAATAGAAAGCTTGACGATGTCTGAGTATG	D_{10}
	D	/5Phos/TGACCATACT CAGACATCGTCA AGCTTTCTATTCGCATCAGCATACTATTCAGGTCTCT	D_{11}
		/5Phos/TAAAAGAGACCTGAATAGTATGCTGATGCGAATAGAAAGCTTGACGATGTCTGAGTATG	D_{12}
	E	/5Phos/TTTAATCTCATCGCATACTCATACTCAGACATCGTCAAGCTTTCTATTCGCATCAGCATACTATTCAGGTCTCT	D_{13}
		/5Phos/CTTAAGAGACCTGAATAGTATGCTGATGCGAATAGAAAGCTTGACGATGTCTGAGTATGAGTATGCGATGAGAT	D_{14}
**Figure S4a**	A	/5Phos/ATGATGAGACCAGCATCTATTTCACATTCACGGTCTCT	D_{15}
		/5Phos/GATTAGAGACCGTGAATGTGAAATAGATGCTGGTCTCA	D_{16}
	B	/5Phos/AATCAGAGTATCAGTAGCTATATGTAGGTACAGGTCGT	D_{17}
		/5Phos/TCATACGACCTGTACCTACATATAGCTACTGATACTCT	D_{18}
**Figure S4d**	A	/5Phos/ATGATGAGACCAGCATCTATTTCACATTCACGGTCTCT	D_{15}
		/5Phos/GATTAGAGACCGTGAATGTGAAATAGATGCTGGTCTCA	D_{16}
	B	/5Phos/AATCAGAGTATC	D_{19}
	C1	/5Phos/TCATATATAGCTACTGATACTCT	D_{20}
	C2	AGCTATAT	D_{21}
**Figure S4f**	A	/5Phos/ATGATGAGACCAGCATCTATTTCACATTCACGGTCTCT	D_{15}
		/5Phos/GATTAGAGACCGTGAATGTGAAATAGATGCTGGTCTCA	D_{16}
	B	/5Phos/AATCAGAGTATC	D_{19}
	C	AGCTATATGTAGGTACAGGTCGT	D_{22}
		/5Phos/TCATACGACCTGTACCTACATATAGCTACTGATACTCT	D_{18}
**Figure S5**		GTATTCCATTCACGGTCTCAAATCC/iBHQ-2dT/CTATTCGCATGACATACG	D_{23}
		CGTATGTCATGCGAATAGAGGATT/iCy3N/GAGACCGTGAATGGAATAC	D_{24}
**Figure 3f**	A	/5Phos/ATGATGAGACCAGCATCTATTTCACATTCACGGTCTCT	D_{15}
		/5Phos/GATTAGAGACCGTGAATGTGAAATAGATGCTGGTCTCA	D_{16}
	B	/5Phos/AATCAGAGTATC/3IABkFQ/	D_{25}
	C	/5Phos/TCATACGACCTGTACCTACATATAGCTACTGATACTCT	D_{18}
		/5Cy3/AGCTATATGTAGGTACAGGTCGT	D_{26}
**Figure 4b**	M2	/5Cy3/AGCTATATGTAGGTACGGTCTCT	D_{27}
		/5Phos/GATTAGAGACCGTACCTACATATAGCTACTGATACTCT	D_{28}

	M3	/5Phos/AATCAGAGTATCTTTTTCGAATAGA	D_{29}
**Figure 4c**	M2	/5Cy3/AGCTATATGTAGGTACGGTCTCT	D_{27}
		/5Phos/GATTAGAGACCGTACCTACATATAGCTACTGATACTCT	D_{28}
	M3	/5Phos/AATCAGAGTATCTTTTTCGAATAGA	D_{29}
	DoS_1	/5BiosG/TTTTTTTTCACCTCTATTCG	D_{30}
**Figure 4d**	M2	/5Cy3/AGCTATATGTAGGTACGGTCTCT	D_{27}
		/5Phos/GATTAGAGACCGTACCTACATATAGCTACTGATACTCT	D_{28}
	M3	/5Phos/AATCAGAGTATCTTTTTCGAATAGA	D_{29}
	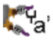	/5Phos/AATCCTCTATTCGCATGAGAATTCCATTCAC	D_{31}
		/5BiosG/TTTTGTGAATGGAATTCTCATGCGAATAGAG	D_{32}
**Figure S8a**	M2	/5Cy3/AGCTATATGTAGGTACGGTCTCT	D_{27}
		/5Phos/GATTAGAGACCGTACCTACATATAGCTACTGATACTCT	D_{28}
	M3	/5Phos/AATCAGAGTATCTTTTTCGAATAGA	D_{29}
	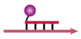	/5Phos/TCATACGACCTGTACCTACATATAGCTACTGATACTCT	D_{18}
		/5Cy3/AGCTATATGTAGGTACAGGTCGT	D_{26}
		/5Phos/ATGATGAGACCAGCATCTATTTCACATTCACGGTCTCT	D_{15}
		/5Phos/GATTAGAGACCGTGAATGTGAAATAGATGCTGGTCTCA	D_{16}
		/5Phos/ATGATGAGACCCTCATCGCATACTCATACTCAGACATCGTCAAGCTTTCTATTCGCATCAGCATACTATTCAGGTCTCT	D_{33}
		/5Phos/GATTAGAGACCTGAATAGTATGCTGATGCGAATAGAAAGCTTGACGATGTCTGAGTATGAGTATGCGATGAGGGTCTCA	D_{34}
**Figure S10a**	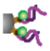	CATAGGTAGAAGTGT/3ATTO488N/	D_{35}
		/5BiosG/ACACTTCTACCTATGTTTTTTTTCACCTCTATTCG	D_{36}
**Figure 5h**	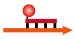	/5Phos/GATTAGAGACCGTACCTACATATAGCTACTGATACTCT	D_{28}
		/5ATTO647NN/AGCTATATGTAGGTACGGTCTCT	D_{38}
	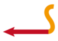	/5Phos/AATCAGAGTATCTTTTTCGAATAGA	D_{29}
**Figure 6**	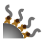	/5BiosG/TTTTTTTTCACCTCTATTCG	D_{30}
	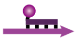	/5Cy3/AGCTATATGTAGGTACGGTCTCT	D_{27}
		/5Phos/GATTAGAGACCGTACCTACATATAGCTACTGATACTCT	D_{28}
	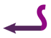	/5Phos/AATCAGAGTATCTTTTTCGAATAGA	D_{29}
	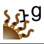	/5BiosG/TTTTTTTTCACCAGTATGAG	D_{37}
	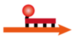	/5ATTO647NN/AGCTATATGTAGGTACGGTCTCT	D_{38}
		/5Phos/ATGAAGAGACCGTACCTACATATAGCTACTACTTGATA	D_{39}
	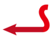	/5Phos/TCATTATCAAGTTTTTTCTCATACT	D_{40}
	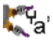	/5Phos/AATCCTCTATTCGCATGAGAATTCCATTCAC	D_{31}
		/5BiosG/TTTTGTGAATGGAATTCTCATGCGAATAGAG	D_{32}

Supplementary Note 1. Development of the conditions for the transient DySS DNA polymerization

Definition of the Weiss Unit to describe the activity of T4 DNA ligase (Promega): 0.01 Weiss Unit [WU] of T4 DNA Ligase is the amount of enzyme required to catalyze the ligation of greater than 95% of 1 µg of λ/HindIII fragments at 16 °C in 20 minutes.
Unit definition to describe the activity of BsaI (*NEB*): One unit is defined as the amount of enzyme required to digest 1 µg of pXba DNA in 1 hour at 37 °C in a total reaction volume of 50 µL.

We investigated the transient DySS polymerization of M1 both at 25 and 37 °C. The restriction enzyme, BsaI, has a higher activity for cleavage at 37 °C, for which we show most systems in the main manuscript. However, for the T4 DNA Ligase, the activity is a balance between higher propensity of sticky ends to transiently bind to each other at low temperature, and reduced enzymatic activity with lower temperature. For comparison, Supplementary Figure 1 displays the transient DNA polymerization at 25 °C fueled with 0.2, 0.4, and 0.6 mM ATP. Compared to the results at 37 °C (Figure 2), the lower activity of the BsaI at 25 °C leads to longer lifetimes and ca. 2000 bp higher $\overline{bp_w}$ in the DySS. For instance the lifetimes at 0.2 mM ATP and 0.4 mM ATP are 1 and 2 days at 25 °C, while they are only ca. 9 h and 18 h at 37 °C, respectively. The higher $\overline{bp_w}$ in the DySS is a result of the lower cutting frequency at 25 °C and thus higher degree of ligation. Correspondingly, the rate of ATP dissipation at 25 °C is lower and the system fueled with 0.6 mM ATP stays active over days. The AGE data calculations were performed according to our previous report.[1]

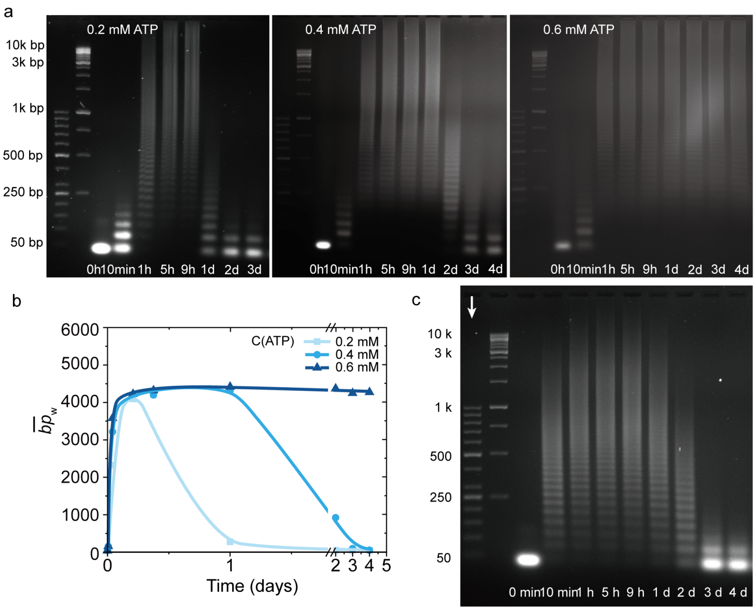

Supplementary Figure 1. ATP-driven transient DNA polymerization of M1. (**a**) Time-dependent AGE (2 wt. %, 90 V, 2 h) for transient DNA polymerization at 25 °C with programmable lifetimes by fueling with 0.2 mM, 0.4 mM, and 0.6 mM ATP. (**b**) The $\overline{bp_w}$ development with time by varying ATP concentrations from 0.2 mM to 0.6 mM. Lines are guides to the eye. (**c**) Time-dependent AGE (2 wt. %, 90 V, 2 h) for transient DNA polymerizations fueled by 1.0 mM ATP at 37 °C showing the starting pockets (at the beginning of the white arrow) for running the gel. Conditions: (**a**) 25 °C, 1× *NEB* CutSmart buffer, 0.05 mM dsDNA tile M1, 0.46 WU µL^{-1} T4 DNA ligase, and 2.5 units µL^{-1} BsaI, varied concentration of ATP.

Supplementary Note 2. Enzyme activity and stability overtime

The enzyme stability of BsaI over time was assessed by the efficacy for the cleavage of a dsDNA tile (annealed from D_3 and D_4) containing a BsaI restriction site in the center after different pre-incubation times of BsaI at 37 °C in CutSmart buffer (0 h, 12 h, 1 day, 2 days, and 3 days). Supplementary Figure 2b-f displays the AGE during restriction after different times during the individual reactions. Supplementary Figure 2g quantifies the time-dependent fractions of the cleaved DNA for different pre-incubation/storage times of BsaI. The highest activity is found for the freshly prepared enzyme solution, while longer pre-incubation shows a gradual loss of activity. The slopes of the curves in Supplementary Figure 2g were calculated in the linear regime (the first 6 h), and normalized to the slope of the cleavage by the enzyme without any pre-incubation to give the relative enzyme activities after different pre-incubation times (Supplementary Figure 2h). After 2 days of incubation at 37 °C, the BsaI has only lost ca. 20 % of its activity. T4 DNA ligase is known to be stable for more than 2 weeks.[1]
The calculations were first performed by Image J. Briefly, rectangular selections were made for every gel band and the grayscale values for each gel band were then measured. Afterwards, the fractions for the cleaved DNA strands in each lane were calculated in percentage ratios and plotted in Supplementary Figure 2g. The slopes of the linear regions (0-8 h) of the cleaved fraction plots were calculated by linear fit via origin. Then, all the slopes were normalized to the slope of cleaved fraction by BsaI without preincubation, which is regarded to have 100 % enzyme activity. The normalized values were plotted as the enzyme activities for different time preincubations.

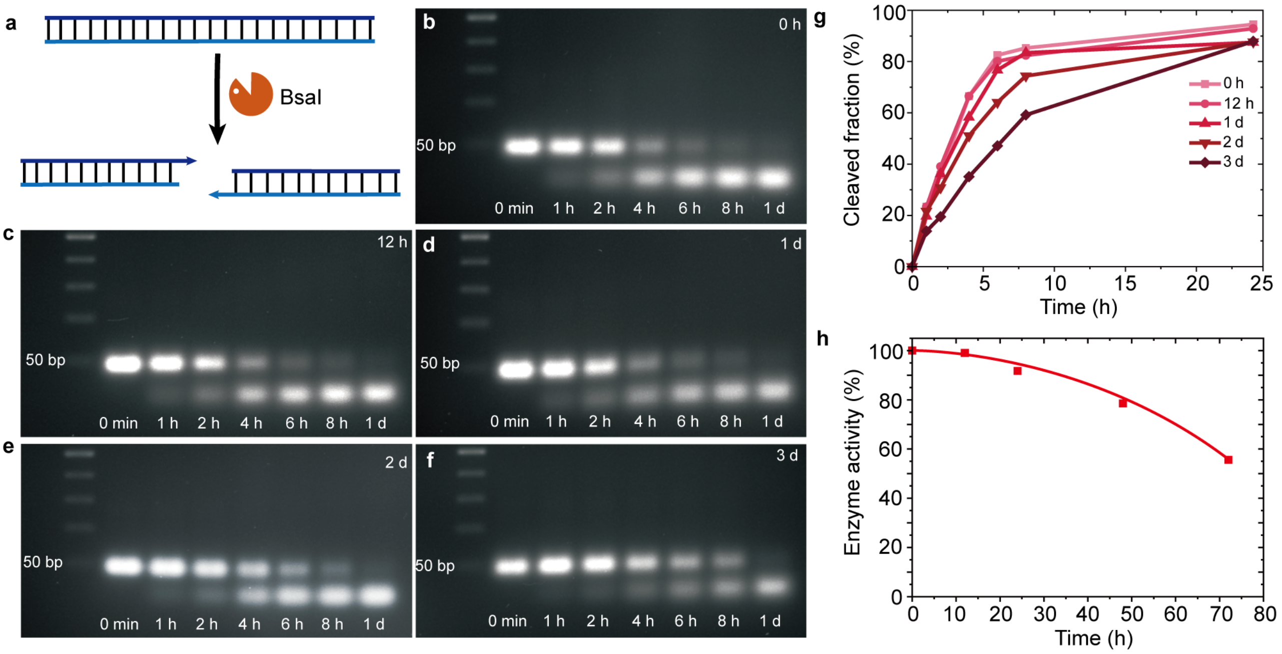

Supplementary Figure 2. BsaI activity and stability after different pre-incubation/storage times. (**a**) Schematic representation of the cleavage of dsDNA by BsaI. (**b-f**) Time-dependent AGE (3 wt. %, 80 V, 2 h) for the dsDNA cleavage by the BsaI with different pre-incubation times at 37 °C (**b**, 0 h; **c**, 12 h; **d**, 1 day; **e**, 2 days; **f**, 3 days). (**g**) Time-dependent fractions of dsDNA cleavage by the restriction enzyme with different time for pre-incubation. (**h**) BsaI activity after varied pre-incubation time. The line is a guide to the eye. Conditions: 37 °C, 1× *NEB* CutSmart buffer, 0.025 mM dsDNA (D_3/D_4), and 1.5 units μL^{-1} BsaI.

Supplementary Note 3. ATP-fueled sequence-defined transient DySS oligomers

Supplementary Figure 3 displays the transient behavior of the DySS oligomers via the inputs of AB, ABC, and ABCDE, respectively. Different outputs can be obtained by changing the combination of inputs. However, for the pentamer ABCDE, statistically expected oligomer ABCD and BCDE are also visible.

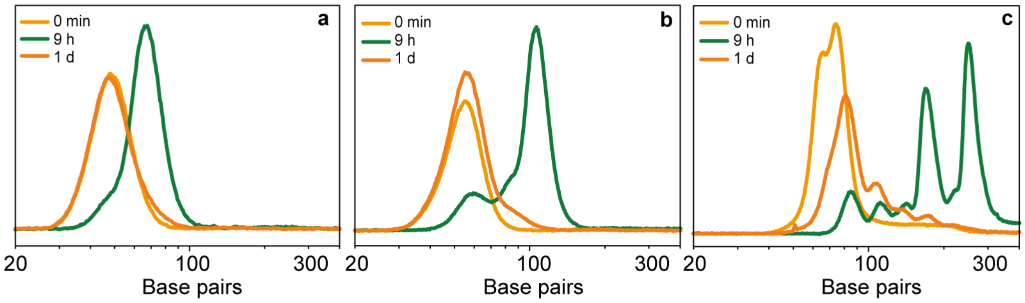

Supplementary Figure 3. Transient DNA polymerization with programmable tiles arrangement quantified from AGE. Gray scale profiles extracted from AGE for the transient polymerization of (**a**) A+B, (**b**) A+B+C and (**c**) A+B+C+D+E. Conditions: 37 °C, 1× *NEB* CutSmart buffer, 0.05 mM dsDNA tiles in total (for each individual system, equal stoichiometry for each tile, tile composition is listed in Supplementary Table 1), 0.92 WU µL^{-1} T4 DNA ligase, 1.0 unit µL^{-1} BsaI, and 0.2 mM ATP.

Supplementary Note 4. Strategies for the design of transient sequence-defined nucleic acid polymers (SfNAPs) that are capable for universal functional group attachment

In this part, we explain the rational for the facile introduction of functional groups into close proximity using easily available end-functionalized ssDNA and only focusing on linear DNA building blocks without pre-encoded junctions such as in T-shaped dsDNA tiles.

Simple alternating transient sequences without functions can be made by combination of two dsDNA tiles via proper programming of the sticky ends (Supplementary Figure 4a-c). However, for this kind of alternating DySS polymer, it is not straightforward to transiently anchor functional groups flexibly (also in close proximity to the ligation site) on the DNA scaffold without extensive redesign. In principle, one may be able to put the functional groups in the middle of the dsDNA tiles via internal nucleobase modifications or intrinsically branched, T-shaped tiles could be used. Nonetheless, such strategies would in any case not allow to put functional groups in close proximity in the DySS, e.g. directly adjacent to each other (due to the space requirement of the restriction site), which is however desirable for efficient FRET signaling or protein and inhibitor pairs.[2-3]

Therefore, we realized a system which has free 5’ and 3’ positions that can be used for universal modification and which allows for close positioning of functional groups in the DySS (Supplementary Figure 4d). The system initially contained 1 dsDNA and 3 ssDNA and is based on a consecutive activation for ligation. One of the sticky ends in the dsDNA monomer A is complementary to a part of the sequence in the ssDNA monomer B, leading to covalent ligation between A and B. Then, the remaining sequence in B is complementary to a part of the sequence in C1, leading to further covalent ligation with C1. The overhang on the other side of C1 is further covalently ligated to the second sticky end on monomer A to make a DySS DNA polymer. C2 is complementary to another part of the sequence in C1 and fills the space between A and B, but leaving a break with free 5’ and 3’ ends. The restriction sites are embedded on both sides of monomer A, enabling recovery of monomer A after the cleavage by BsaI. Critically, the hybridization length between B and C1 and between C1 and C2 are only 8 bp, which have T_ms of ca. 33 °C. Their assembly is conditional on the ligation taking place. After the cleavage, B, C1, and C2 are separated from each other. This strategy indeed allows transient and alternating ATP-driven DySS DNA polymers with functionalizable 5’ and 3’ ends that end up in adjacent positions (Supplementary Figure 4e,h). However, the overall length of the alternating DySS DNA polymer is comparably short compared to the strategy of using 2 dsDNA monomers in Supplementary Figure 4a. We attribute this to the inefficient ligation of the tiles by ligating 1 dsDNA with 3 ssDNA for the growth of the DNA chain, for which there is more ligation steps than the ligation between 2 dsDNA monomers.

To reduce this unwanted effect, we simply elongated C1 and C2 to allow their hybridization into a stable dsDNA tile, i.e. monomer C, before the initiation of the transient system (Supplementary Figure 4f). Then, the system changes to 2 dsDNA with 1 ssDNA. This indeed promotes a beneficial increase in the molecular weight and the transient polymerization of this alternating DNA polymer is comparable to that for the alternating DNA polymer formed by 2 dsDNA monomer, while now offering two adjacent 3’ and 5’ sites for functionalization (Supplementary Figure 4g,i). Critically, those sites are only brought in close proximity using the ATP-driven dynamic system, as this allows to link B to A, which then serves as template to link C to A. In absence of ATP, the tiles remain disassembled due to the low T_m of the overhangs (see Supplementary Note 6).

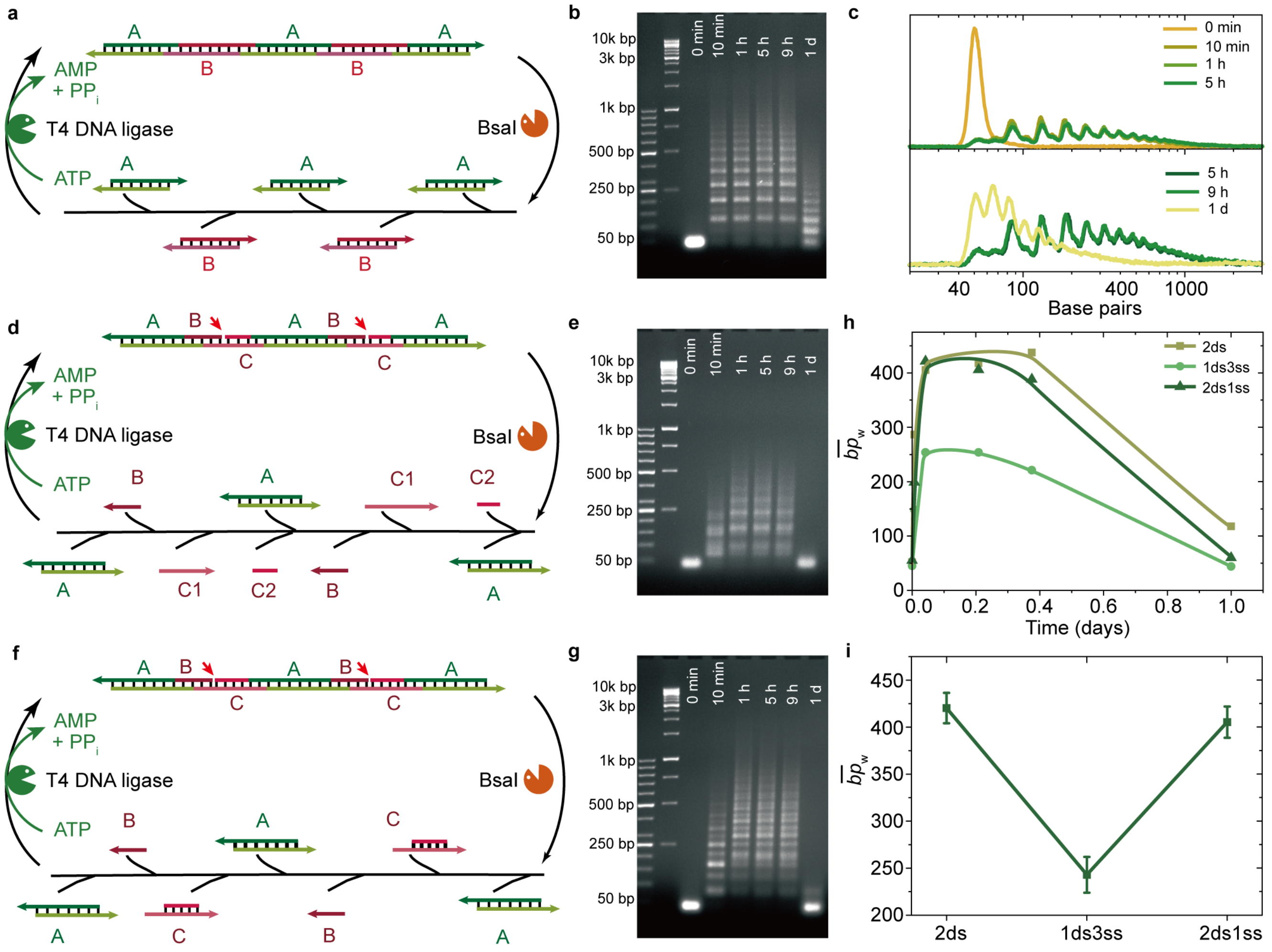

Supplementary Figure 4. Strategy towards sequence-defined DySS functionalized nucleic acid polymers (SfNAPs). (**a**) Representation of ATP-driven transient, alternating DySS DNA polymer by 2 dsDNA monomers, A (annealed from D_{15} and D_{16}) and B (D_{17} and D_{18}). (**b**) Time-dependent AGE (2 wt.%, 90 V, 2 h) of the transient DNA polymerization by 2 dsDNA tiles fueled with 0.2 mM ATP. (**c**) Gray scales profiles extracted from AGE of the transient DNA polymerization with 2 dsDNA quantifying the transient shift of molecular weight, which is used to calculate the mass-weighted average chain length ($\overline{bp}_w$) for each kinetic aliquot. (**d**) Schematic illustration of an ATP-driven transiently alternating DNA polymer from 1 dsDNA and 3 ssDNA tiles. (**e**) Time-dependent AGE (2 wt.%, 90 V, 2 h) of the transient DNA polymerization by 1 dsDNA and 3 ssDNA fueled with 0.2 mM ATP. (**f**) Schematic illustration of an ATP-driven transiently alternating DNA polymer from 2 dsDNA and 1 ssDNA. (**g**) Time-dependent AGE (2 wt.%, 90 V, 2 h) of the transient DNA polymerization by 2 dsDNA and 1 ssDNA fueled with 0.2 mM ATP. (**h**) The $\overline{bp}_w$ development with time by different polymerization mechanisms for the alternating DNA polymers. Lines are guides to the eye. (**i**) Averaged $\overline{bp}_w$ for the alternating DNA polymer with different mechanisms. Error bars are standard deviations from duplicate experiments. Conditions: (**a**) 37 °C, 1× *NEB* CutSmart buffer, 8.0 µM A (D_{15} and D_{16}), 8.0 µM B (D_{17} and D_{18}), 0.92 WU µL^{-1} T4 DNA ligase, and 1.0 unit µL^{-1} BsaI, and 0.24 mM ATP. (**d**) 37 °C, 1× *NEB* CutSmart buffer, 8.0 µM A (D_{15} and D_{16}), 8.0 µM B (D_{19}), 8.0 µM C1 (D_{20}), 8.0 µM C2 (D_{21}), 0.92 WU µL^{-1} T4 DNA ligase, 1.0 unit µL^{-1} BsaI, and 0.24 mM ATP. (**f**) 37 °C, 1× *NEB* CutSmart buffer, 8.0 µM A (D_{15} and D_{16}), 8.0 µM B (D_{19}), and 8.0 µM C (D_{18} and D_{22}), 0.92 WU µL^{-1} T4 DNA ligase, 1.0 unit µL^{-1} BsaI, and 0.24 mM ATP.

Supplementary Note 5. In-strand modifications close to the restriction site

Although we did not screen for all possible combinations of in-strand modifications using non-native nucleobases, we explored whether anchoring some functional groups into the sequences downstream to the cutting site of the restriction site would impose problems on the functioning of the enzymatic reaction network. The dsDNA tile in Supplementary Figure 5 shows an example, bearing a Cy3 and a BHQ1 in downstream of the cleavage site. Indeed the BsaI restriction is fully inhibited (no change in AGE) which underscores the relevance of the methods described in Supplementary Note 4 to integrate functionality into the DySS systems.

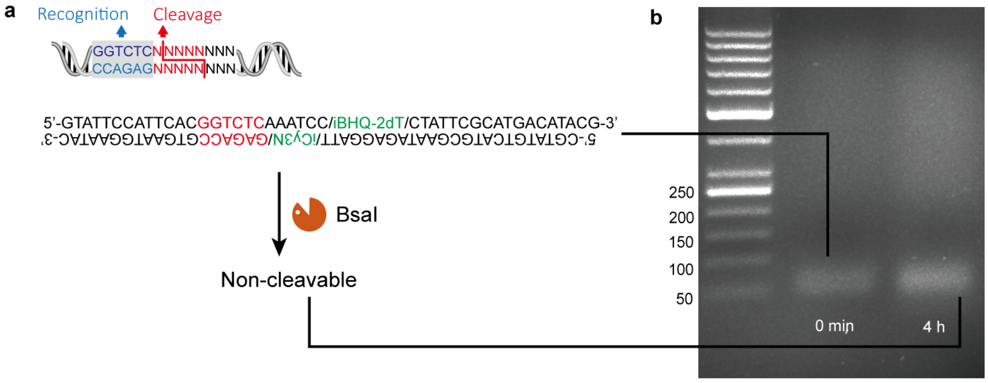

Supplementary Figure 5. Attempted cleavage of the DNA strands with in-strand Cy3 and BHQ1 modifications close to the restriction site. (a) Schematic representation of BsaI recognition site and cleavage position as well as the schematic representation of BsaI cleavage for dsDNA with fluorophore and quencher modifications around its cleavage site. (b) AGE of the dsDNA before and after cleavage by BsaI show identical migration and hence absence of restriction. Condition: 37 °C, 1× *NEB* CutSmart buffer, 0.025 mM dsDNA (D_{23} and D_{24}), 1.5 units μL^{-1} BsaI, 4 h.

Supplementary Note 6. Nupack simulation of the design with 2 dsDNA and 1 ssDNA

The paring and melting of the DNA strands in the design of the transient SfNAP (Supplementary Figure 4f) with free 5′ and 3′ ends for modifications was further analyzed by Nupack. Supplementary Figure 6a gives the overall information of the five DNA strands used for the system design. Under the experimental condition, as expected, strand1 and strand2 hybridize and give rise to dsDNA monomer A and strand3 and strand4 make another dsDNA monomer C. B and C are separated from each other in the non-fueled state (Supplementary Figure 6b). Supplementary Figure 6c summarizes the overall concentrations of each hybridized duplex and non-hybridized ssDNA.

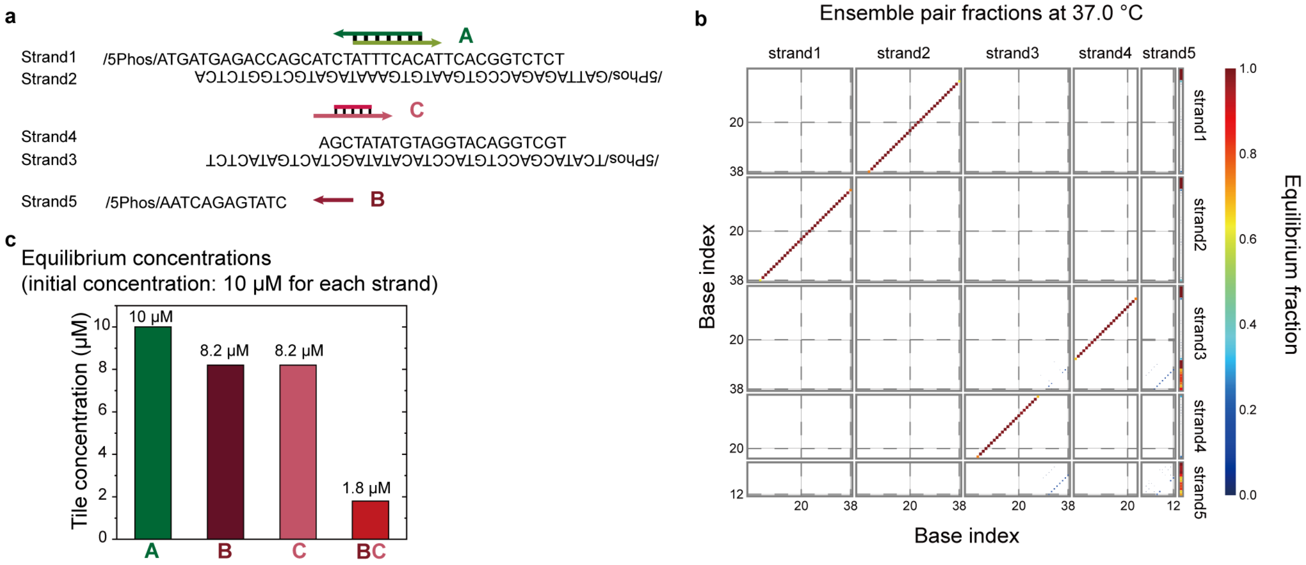

Supplementary Figure 6. Nupack simulation for pairing and melting of the multicomponent systems discussed in Supplementary Figure 4f. (**a**) Sequence information for the ds and ssDNA for the multicomponent system. (**b**) Nupack simulation association map of the pairing and hybridization of these five strands. (**c**) Concentration of each strand at equilibrium condition.

Supplementary Note 7. Comparison of the transient sequence-defined nucleic acid polymers (SfNAP) with and without modifications

Following the design of the SfNAPs with 2 dsDNA and 1 ssDNA (Figure 3f and Supplementary Figure 4f), we here report whether the incorporated fluorophore (Cy3) and quencher (IABkFQ) have effects on the operation of the chemical reaction network. Indeed, when comparing the temporal developments in AGE of all systems at the different ATP concentrations, no significant differences are discernible, independent on the presence or absence of chemical modifications (Supplementary 7a,b). Refueling is also possible as additionally depicted in Supplementary Figure 7e.

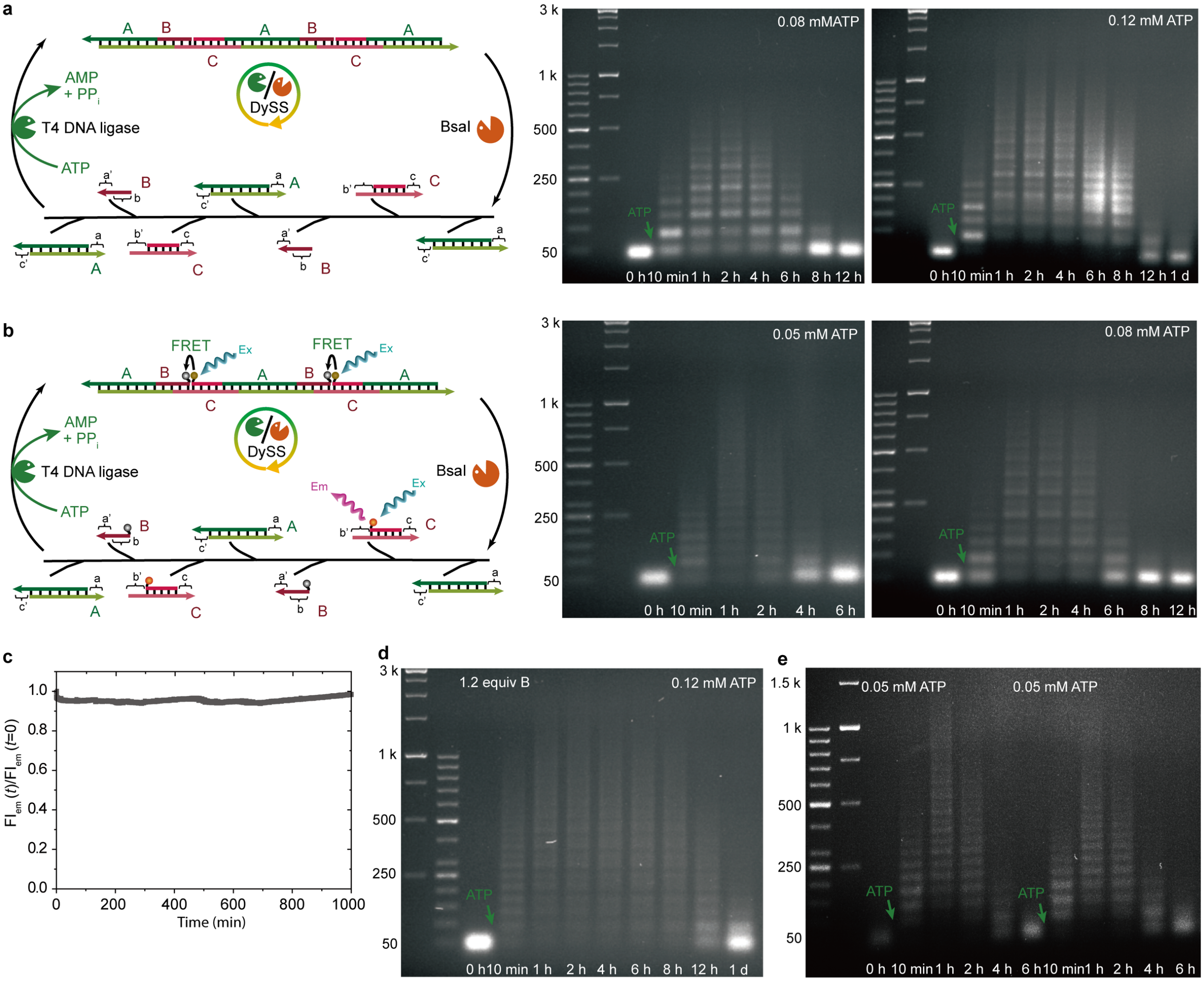

Supplementary Figure 7. Analysis of the transient SfNAP with and without fluorophore/quencher modifications. (**a**) Transient polymerization of 2 dsDNA and 1 ssDNA without any fluorophore and quencher for the alternating DNA polymer fueled by 0.08 mM and 0.12 mM ATP. (**b**) Transient polymerization of 2 dsDNA and 1 ssDNA with Cy3 and IABkFQ for the alternating DNA polymer by 0.05 mM and 0.08 mM ATP. (**c**) Control experiment for transient FRET signaling without ATP, shows absence of FRET and hence no polymer formed. (**d**) Transient polymerization of 2 dsDNA and 1 ssDNA with Cy3 and IABkFQ for the alternating DNA polymer fueled by 0.12 mM ATP, for which 1.2 equivalents of the ssDNA-IABkFQ were added. (**e**) Repeated fuel addition of the transient FRET SfNAP by 0.05 mM ATP. Conditions: (a) 37 °C, 1× *NEB* CutSmart buffer, 8.0 µM A (D_{15} and D_{16}), 8.0 µM B (D_{19}), and 8.0 µM C (D_{22} and D_{18}), 0.92 WU µL^{-1} T4 DNA ligase, 1.0 unit µL^{-1} BsaI, and ATP. (**b**) 37 °C, 1× *NEB* CutSmart buffer, 8.0 µM A (D_{15} and D_{16}), 8.0 µM C (D_{18} and D_{26}), 8.0 µM B (D_{25}), 0.92 WU µL^{-1} T4 DNA ligase, 1.0 unit µL^{-1} BsaI, and ATP. (**d**) Performed the same as (**b**) but 1.2 equivalents of ssDNA-B (D_{25}) was added. (**e**) Performed the same as (**b**) but fueled twice with 0.05 mM ATP.

Supplementary Note 8. Effective multivalent recognition in transient SfNAPs by engineering side chain interactions.

We used three static SfNAPs (P1-$d1_b$, P2-$d2_b$, and P3-$d3_b$) with different branch distances of 38 bp, 76 bp, and 117 bp (synthesized by ATP-fueled ligation, Supplementary Figure 8a) to investigate what kind of distance between the side chains is needed to allow for efficient multivalent cooperative binding with a colloidal target at a specific interaction strength of an individual overlapping ssDNA overhang (L_c, here 8 bp, see Figure 4c). The static SfNAPs were labeled with Cy3 to directly visualize the multivalent binding between the SfNAPs and the colloids. The interacting side chain is complementary to the ssDNA on the DoS_1-modified magnetic colloid. Supplementary Figure 8b-d depict the length distributions of the three SfNAPs, from which we also calculated the number of branches on the SfNAPs (P_w). Importantly, all three SfNAPs have a similar P_w distribution and average, $\overline{P}_w$, thus allowing to discern the minimum branching density needed to reach efficient multivalent interactions. To this end, the SfNAPs, P1-$d1_b$, P2-$d2_b$, and P3-$d3_b$, were separately mixed with DoS_1-modified colloids and the extent of colloid assembly was qualified by CLSM. Only P1-$d1_b$ can induce efficient multivalent DNA wrapping on the colloidal surface, leading to colloid self-assembly due to chain bridging. An increase of the branch distance to 76 bp in P2-$d2_b$ only induces very limited colloid self-assembly and the fluorescent shell on the colloid surface is very weak. An even further increase of the branch distance to 117 bp in P3-$d3_b$ disallows assembly and a fluorescent shell cannot be observed, hence demonstrating the absence of sufficient multivalent interactions (Supplementary Figure 8f). Based on these screening experiments, we employed the structural design of P1-$d1_b$ for all investigations within the main body of the manuscript.

Supplementary Figure 8g shows the AGE result of the transient SfNAPs from TCA in Figure 5b. In order to check whether there is selective binding of differently long DySS SfNAPs onto the colloids during the ATP-fueled process, we further investigated the length distributions during transient polymerizations of P1-$d1_b$ with and without DoS_1-functionalized colloids. AGE of both transient polymerizations does not indicate any obvious differences of the SfNAP distributions in these two cases (Supplementary Figure 8h).

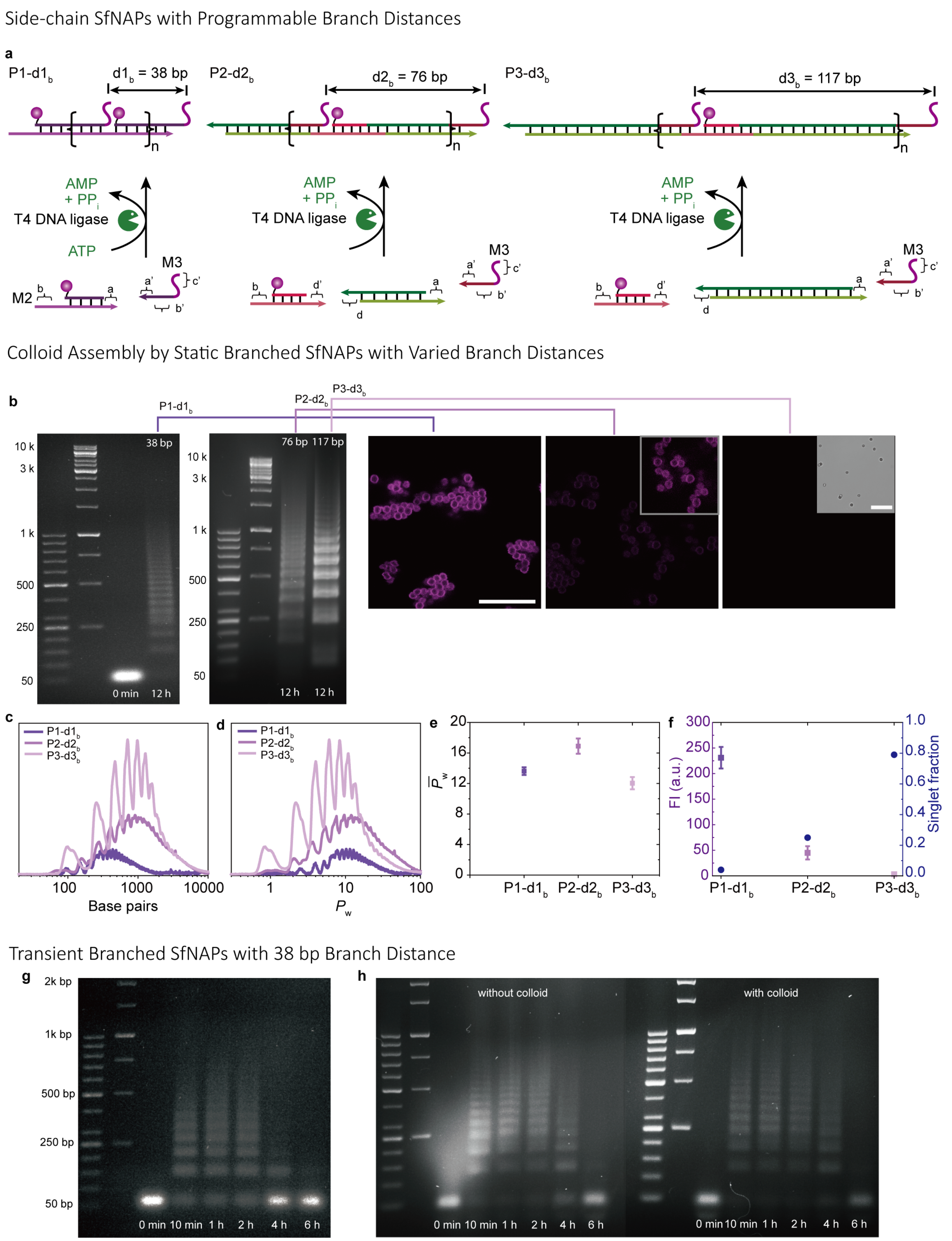

Supplementary Figure 8. Effects of the branch distance in SfNAPs on the multivalent DNA wrapping and SfNAP-mediated colloidal self-assembly. (**a**) Schematic representation of the synthesis of P1-$d1_b$ (polymerization of dsDNA-Cy3 (D_{27} and D_{28}), and ssDNA-branch (D_{29})), P2-$d2_b$ (polymerization of dsDNA from D_{15} and D_{16}, Cy3-labeled dsDNA annealed from D_{18} and D_{26}, and ssDNA-branch (D_{29})), and P3-$d3_b$ (polymerization of dsDNA from D_{33} and D_{34}, Cy3-labeled dsDNA annealed from D_{18} and D_{26}, and ssDNA-branch (D_{29})). (**b**) AGE results for the SfNAPs and static assembly studies of these SfNAPs with DoS_1-modified colloids characterized by CLSM (consecutively measured at exactly the same settings, the inserted picture

was optimized by brightness and contrast for better viewing). Scale bars, 10 µm. (**c**) Molecular weight distributions of the SfNAPs plotted from the gray scales in AGE. (**d**) P_w distributions for these SfNAPs calculated from the molecular weight distributions. (**e**) $\overline{P}_w$ for the SfNAPs. Error bars are standard deviations from duplicate experiments. (**f**) Fluorescence intensity (consecutively measured by CLSM at exactly the same settings) for the DNA shell on the colloidal surface after the SfNAPs were wrapped to the colloidal surface and its corresponding singlet fraction of the colloid after colloid assembly. Error bars are standard deviations of random 50 measurements of the colloids. (**g**) Time-dependent AGE (2 wt.%, 90 V, 2 h) results of the transient DNA polymerization from TCA in Figure 5b. (**h**) Time-dependent AGE (2 wt.%, 90 V, 2 h) results of the transient DNA polymerization with and without colloid, fueled with 50 µM ATP. Conditions: (**a**) P1-$d1_b$, 37 °C, 1× *NEB* CutSmart buffer, 10.0 µM dsDNA-Cy3 (D_{27} and D_{28}), 12.0 µM ssDNA-branch (D_{29}), 0.92 WU μL^{-1} T4 DNA ligase, and 0.1 mM ATP, 12 h. P2-$d2_b$ and P3-$d3_b$, 37 °C, 1× *NEB* CutSmart buffer, 10.0 µM Spacer tile (dsDNA from D_{15} and D_{16} for P2-$d2_b$, dsDNA from D_{33} and D_{34} for P3-$d3_b$), 10.0 µM Cy3 labeled dsDNA (D_{18} and D_{26}), 12.0 µM ssDNA-branch (D_{29}), 0.92 WU μL^{-1} T4 DNA ligase, and 0.2 mM ATP, 12h. (b) 0.167 mg mL^{-1} DoS_1-modified colloids were separately added to the as-synthesized solutions of SfNAPs. (**g**) 37 °C, 1× *NEB* CutSmart buffer, 10.0 µM dsDNA-Cy3 (D_{27} and D_{28}), 12.0 µM ssDNA-branch (D_{29}), 0.92 WU μL^{-1} T4 DNA ligase, 1.0 units μL^{-1} BsaI, ca. 0.167 mg mL^{-1} DoS_1 modified colloids, and 50 µM ATP. (h) Same condition as (**g**) except that the two reactions were divided from the same batch and only one batch was added with 0.167 mg mL^{-1} DoS_1-modified colloids.

Supplementary Note 9. Programmable lifetimes in TCA

Supplementary Figure 9a and b show the CLSM images of TCA fueled by 0.1 and 0.2 mM ATP, respectively, which changes the lifetime of the TCA from 8 h to 16 h. Simultaneously, the AGE results of the transient SfNAPs isolated during the TCA show corresponding lifetimes.

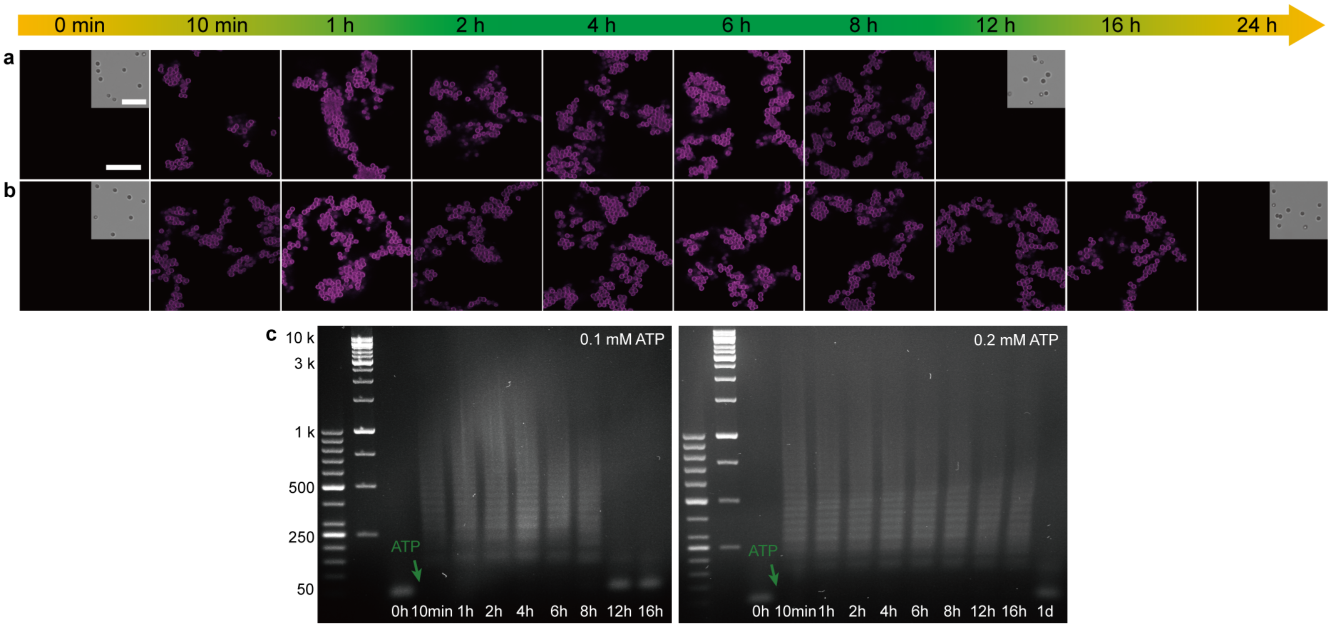

Supplementary Figure 9. Transient colloid assembly (TCA) fueled with ATP. Time-dependent CLSM images of TCA fueled with (**a**) 0.1 and (**b**) 0.2 mM ATP. Scale bars, 10 µm. (**c**) Time-dependent AGE (2 wt.%, 90 V, 2 h) results of TCA fueled with 0.1 and 0.2 mM ATP. Conditions: 37 °C, 1× *NEB* CutSmart buffer, 10.0 µM dsDNA-Cy3 (D_{27} and D_{28}), 12.0 µM ssDNA-branch (D_{29}), 0.92 WU µL^{-1} T4 DNA ligase, 1.0 unit µL^{-1} BsaI, ca. 0.167 mg mL^{-1} DoS_1 modified colloids, and 0.1 or 0.2 mM ATP.

Supplementary Note 10. Control experiments for TCA with dye-labeled colloids

To make the freely dispersed colloids visible before the TCA, the docking strand on the colloidal surface was elongated to harbor an additional ATTO 488-labeled ssDNA, while still presenting the same overhang on the surface as for the standard DoS$_1$-functionalized colloids. Subsequent addition of an ATTO 647-labelled, complementary SfNAP system leads to wrapping and assembly and dual color observations upon ATP addition, whereby the ATTO 647 channel shows transient fluorescence. Transient color coding of the colloid is achieved by the TCA (Supplementary Figure 10 a-c). Supplementary Figure 10d shows the AGE result of the transient SfNAPs from TSP in Figure 5d.

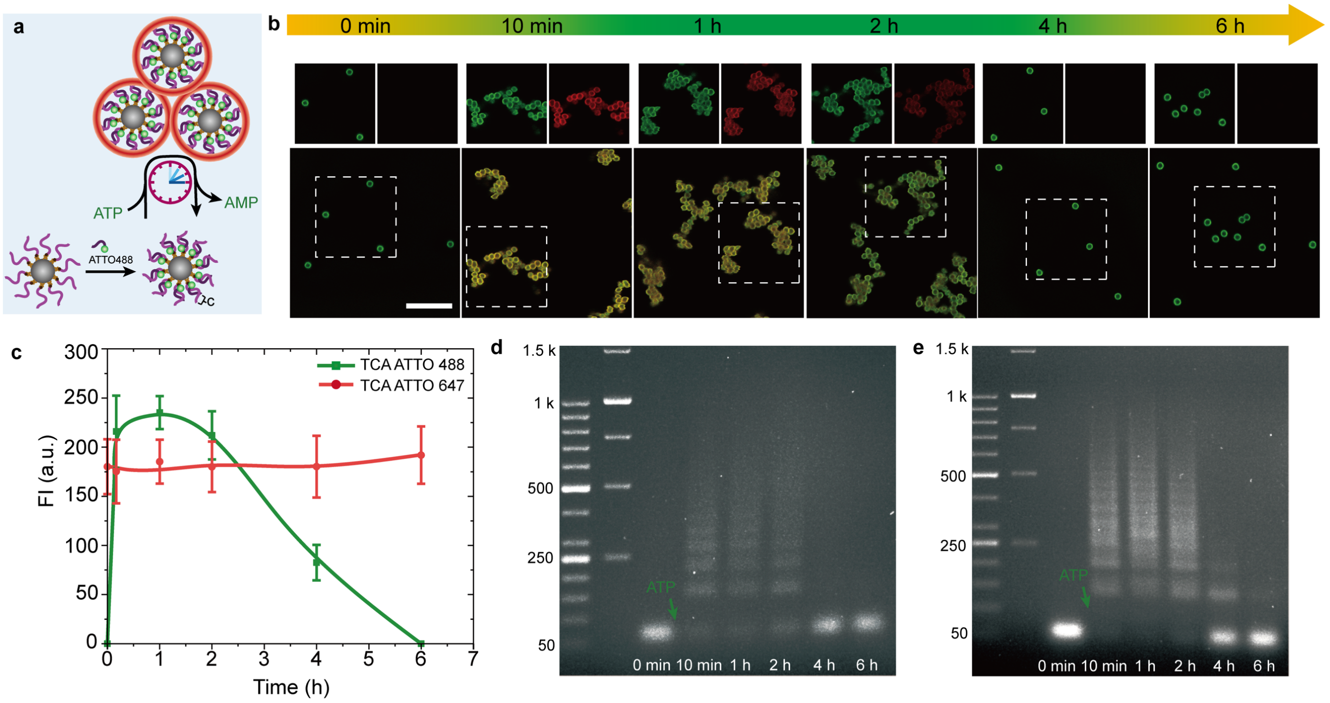

Supplementary Figure 10. Transient colloid assembly with dual color encoding. (**a**) Schematic representation of the chemical fuel driven TCA with pre-immobilized fluorophore on surface. (**b**) CLSM of the TCA over time. (**c**)Time-dependent fluorescence intensity of the fluorescent shell for TCA. Scale bar, 10 µm. Error bars are standard deviations of random 50 measurements of the colloids. Lines are guides to the eye. (**d**) Time-dependent AGE (2 wt.%, 90 V, 2 h) for the TCA. (**e**) Time-dependent AGE of the transient DNA surface polymerization from the TSP system in Figure 5d. Conditions: (**a**) 37 °C, 1× *NEB* CutSmart buffer, 10.0 µM dsDNA-Cy3 (D_{27} and D_{28}), 12.0 µM ssDNA-branch (D_{29}), 0.92 WU µL^{-1} T4 DNA ligase, 1.0 unit µL^{-1} BsaI, ca. 0.167 mg mL^{-1} ATTO 488/L-DoS$_1$ modified colloids, and 50 µM ATP. (**e**) 37 °C, 1× *NEB* CutSmart buffer, 10.0 µM dsDNA-Cy3 (D_{27} and D_{28}), 12.0 µM ssDNA-branch (D_{29}), 0.92 WU µL^{-1} T4 DNA ligase, 1.0 unit µL^{-1} BsaI, ca. 0.167 mg mL^{-1} dsDNA (D_{31} and D_{32}) modified colloids, and 50 µM ATP.

Supplementary Note 11. Integration of secondary tiles and their randomization during TSP of DNA SfNAP brushes on colloids

The following data shows the full temporal course of the data provided briefly in Figure 5i,j.

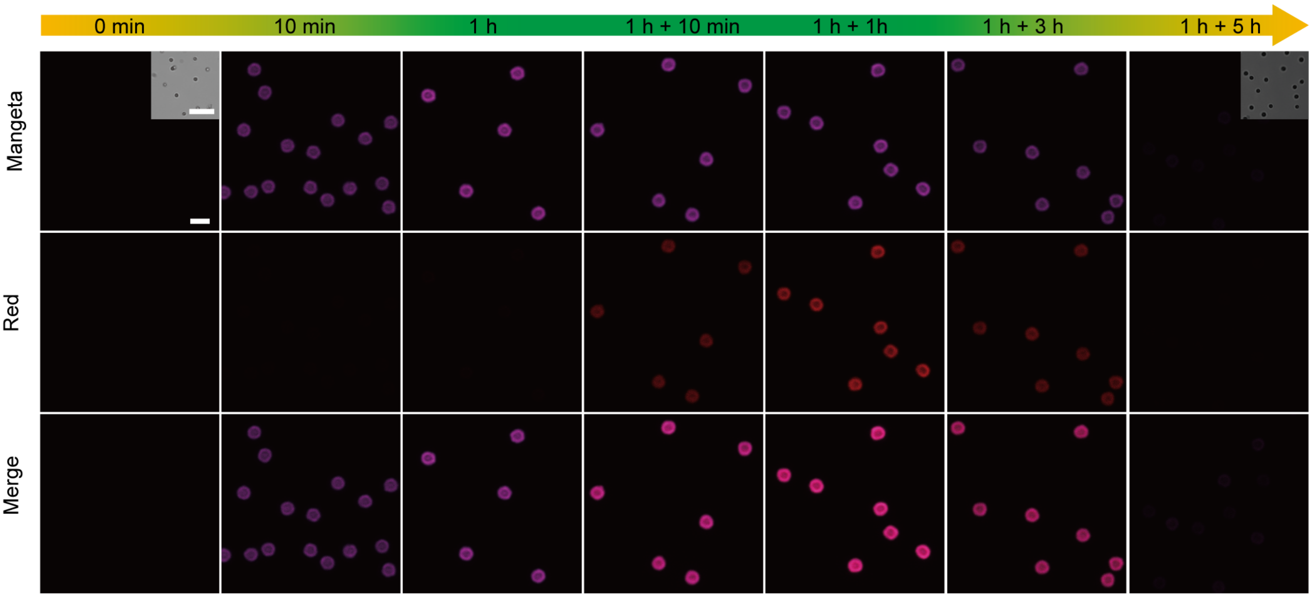

Supplementary Figure 11. Transient DNA surface polymerization (TSP) on colloidal surfaces and dynamic tile integration and randomization. Scale bar, 2 µm; inserted, 10 µm. Conditions: 37 °C, 1× *NEB* CutSmart buffer, 10.0 µM dsDNA-Cy3 (D_{27} and D_{28}), 12.0 µM ssDNA-branch (D_{29}), 0.92 WU µL^{-1} T4 DNA ligase, 1.0 unit µL^{-1} BsaI, ca. 0.167 mg mL^{-1} dsDNA (D_{31} and D_{32}) modified colloids, and 50 µM ATP; after 1 h of TSP, 3 µM ATTO 647-labelled dsDNA (D_{28} and D_{38}) together with 3.6 µM ssDNA-branch (D_{29}) were added to the system.

Supplementary Reference

1. L. Heinen, A. Walther, Programmable dynamic steady states in ATP-driven nonequilibrium DNA systems. *Sci. Adv.* **5**, eaaw0590 (2019).
2. R. M. Clegg, A. I. Murchie, A. Zechel, D. Lilley, Observing the helical geometry of double-stranded DNA in solution by fluorescence resonance energy transfer. *Proc. Natl. Acad. Sci. U.S.A.* **90**, 2994-2998 (1993).
3. B. M. Janssen, W. Engelen, M. Merkx, DNA-directed control of enzyme–inhibitor complex formation: a modular approach to reversibly switch enzyme activity. *ACS Synth. Biol.* **4**, 547-553 (2014).
